# Klotho in diabetes mellitus: research progress and clinical implications

**DOI:** 10.3389/fendo.2025.1740415

**Published:** 2025-12-08

**Authors:** Tingyuan Zhu, Songfeng Zhao, Ning Lu

**Affiliations:** 1 Department of Clinical Laboratory, The First People’s Hospital of Xiaoshan District, Hangzhou, China; 2 Department of Endocrinology, The First People’s Hospital of Xiaoshan District, Hangzhou, China

**Keywords:** Klotho, diabetes mellitus, diabetic complications, biomarker, gene polymorphism, islet function

## Abstract

Diabetes mellitus (DM) and its complications pose a major global health burden, while currently used biomarkers such as HbA1c and microalbuminuria remain limited for early diagnosis and individualized management. Klotho, originally identified as an anti-aging protein, has recently gained increasing attention due to its roles in mineral metabolism, oxidative stress regulation, inflammation, and fibrosis. This review discusses the biological functions of Klotho and its involvement in the pathogenesis of DM, with a particular emphasis on its regulatory mechanisms in glucose metabolism and oxidative stress. We comprehensively summarize recent findings on the role of Klotho in diabetic complications, including diabetic kidney disease, retinopathy, neuropathy, and cardiovascular disease, and highlight evidence on Klotho gene polymorphisms that influence susceptibility to DM and its complications across different populations. Furthermore, we analyze the limitations of current studies, including inconsistent findings on circulating Klotho levels, lack of standardized detection methods, and insufficient large-scale clinical trials. Finally, this review explores the potential of Klotho as both a biomarker and therapeutic target, and outlines future research directions focusing on standardization, mechanistic studies, and translational applications to advance precision medicine in diabetes management.

## 1 Introduction

Diabetes mellitus (DM) is a group of metabolic diseases characterized by chronic hyperglycemia, which is caused by defects of insulin secretion, dysfunction of insulin action or both (1, 2). With the aging of the global population, lifestyle changes and the prevalence of obesity, the prevalence of diabetes continues to rise, which has become a major public health problem that seriously threatens human health (3, 4). According to data from the International Diabetes Federation (IDF) in 2021, about 537 million adults worldwide have diabetes, which is expected to rise to 643 million by 2030 and more than 700 million by 2045 (5). Diabetes not only significantly reduces the quality of life of patients, but also brings a heavy economic burden to the society and the medical system.

The harm of diabetes lies not only in the increase of blood glucose itself, but also in its long-term complications. Diabetic kidney disease (DKD), diabetic retinopathy (DR), diabetic neuropathy (DN), cardiovascular disease (CVD) and diabetic foot are all important causes of disability and death (6–9). Existing clinical markers (such as glycated hemoglobin HbA1c, urinary albumin, glomerular filtration rate GFR, etc.) have certain value in disease diagnosis and risk prediction, but there are obvious limitations in identifying early stages of disease and individualized intervention (10–12). For example, the presence of microalbuminuria often suggests that substantial damage has occurred to the kidney, and HbA1c cannot reflect blood glucose fluctuations or the risk of individualized complications in patients. Therefore, exploring new predictors and therapeutic targets is one of the key directions of diabetes research. In this context, Klotho protein has gradually attracted attention. In 1997, Kuro-o et al. first reported that the mutation of Klotho gene in mice could lead to a series of phenotypes similar to premature aging, including growth retardation, arteriosclerosis, osteoporosis and shortened life span, thus revealing the important role of Klotho in the regulation of aging (13, 14).Subsequent studies have found that Klotho is not only an anti-aging molecule, but also involved in mineral metabolism, energy homeostasis, oxidative stress regulation, apoptosis and fibrosis (15, 16).

The Klotho family mainly includes α-Klotho, β-Klotho and γ-Klotho. Among them, α-Klotho is most closely related to diabetes and its complications. α-Klotho is highly expressed in the distal convoluted tubules of the kidney, and can be used as a membrane-bound protein to participate in fibroblast growth factor 23 (FGF23) signal transduction and regulate calcium and phosphorus metabolism; it can also be cleaved to form soluble Klotho (sKlotho), which is released into the blood and plays a systemic role, including regulating ion channels, inhibiting insulin/IGF-1 signal, reducing oxidative stress and inflammation (16, 17). As a co-receptor of FGF19 and FGF21 signaling pathways, β-Klotho is involved in the regulation of glucose and lipid metabolism, and plays an important role in the maintenance of insulin sensitivity and energy metabolism (18–20). Therefore, α-Klotho and β-Klotho represent two important pathways for the protection mechanism of diabetic complications and the regulation of metabolic homeostasis, respectively.

The current research on Klotho in diabetes mainly focuses on the following three levels. First: A large number of basic and animal experiments have shown that Klotho protects target organs through anti-oxidation, anti-inflammatory, anti-fibrosis and other effects. For example, in a diabetic nephropathy model, Klotho reduced podocyte apoptosis by activating Nrf2 signaling; in diabetic retinopathy, Klotho can alleviate oxidative stress and inflammatory damage; in the cardiovascular system, Klotho exerts cardiovascular protection by inhibiting NLRP3 inflammasome and Wnt/β-catenin pathway (21–23). Second: A growing number of clinical studies have shown that serum or urine Klotho levels are closely related to the risk and progression of diabetes and its complications. For example, low levels of Klotho are associated with DKD, DR, and cardiovascular events, but the results of different studies are heterogeneous, suggesting the importance of detection methods and population characteristics (24, 25). Third: Animal experiments and early exploratory studies suggest that exogenous supplementation of Klotho protein, Klotho-derived peptides, and up-regulation of endogenous Klotho by drugs or genetic engineering may improve diabetes-related organ damage. At the same time, β-Klotho-related FGF21 analogue drugs have entered the clinical trial stage, providing new possibilities for the treatment of diabetes. In summary, Klotho from basic research to clinical transformation, is becoming a research hotspot in the field of diabetes. Its research progress in the three levels of mechanism, marker and treatment provides a new opportunity for the accurate diagnosis, treatment and individualized intervention of diabetes. This article will systematically summarize the research progress of Klotho in diabetes and its complications in recent years, focusing on its molecular mechanism, clinical application potential and future development direction.

## 2 Biological overview of Klotho

### 2.1 Members and structure of Klotho family

The Klotho protein family belongs to the type I transmembrane protein, which mainly includes three subtypes: α-Klotho, β-Klotho and γ-Klotho. The three have certain homology in structure, but there are differences in distribution and function (16, 26). α-Klotho is the most widely studied subtype, which is mainly expressed in renal distal tubular epithelial cells, cerebral choroid plexus and parathyroid glands. It consists of a transmembrane region and two repeated glycosidase-like domains (KL1, KL2). Through metalloproteinase-mediated cleavage, membrane-bound α-Klotho can be released into blood or urine to form soluble Klotho (sKlotho). Membrane-bound α-Klotho is mainly involved in mineral metabolism as a co-receptor of FGF23, while soluble α-Klotho plays a systematic role in anti-aging and metabolic regulation (15). β-Klotho is mainly distributed in the liver, adipose tissue and pancreas. It is an indispensable co-receptor of FGF19 and FGF21 signaling pathways. It regulates glucose and lipid metabolism, bile acid synthesis and energy balance by binding to receptors such as FGFR1 c, and shows potential application value in the treatment of metabolic diseases (27, 28).However, γ-Klotho is mainly expressed in the reproductive system and some tumor tissues. Its interaction with the FGF family is still under study, and the evidence of its direct relationship with diabetes is still limited.

Collectively, these findings highlight that Klotho−derived peptides represent simplified pharmacologic mimetics of native Klotho, targeting FGFRs, TRP channels, and TGF−β receptors to reproduce key cytoprotective and metabolic functions of the full−length protein (29). Other Klotho peptides, such as KP6 and KP7, are derived from conserved regions within KL2 and demonstrate high affinity for TRPV5 and Na^+^/K^+^−ATPase, modulating calcium handling and cellular stress responses (30). Certain peptides have also been shown to bind TGF−β receptor II, reproducing the antifibrotic effects of soluble Klotho by preventing Smad2/3 activation. These binding properties explain why Klotho−derived peptides exhibit protective effects in animal models of kidney injury, diabetic vasculopathy, and β−cell dysfunction. Several Klotho−derived therapeutic peptides have been developed based on functional motifs within the KL1 and KL2 domains (31, 32). Most bioactive Klotho peptides originate from short amino−acid stretches in the KL1 domain that retain the ability to modulate ion channels, attenuate oxidative stress pathways, or interfere with growth factor receptor activation. Representative examples include the KL1−based peptide KP1 (also referred to as Klotho−derived peptide 1), which binds to FGFR1c and partially mimics the co−receptor activity of full−length Klotho, thereby enhancing FGF21 signaling and exerting metabolic benefits *in vivo*.

### 2.2 Physiological function of α-Klotho

Since Kuro-o et al. (1997) found that Klotho-deficient mice showed a premature aging phenotype, the study of α-Klotho has developed rapidly. The physiological function of α-Klotho covers many aspects. First, in terms of mineral metabolism and calcium and phosphorus homeostasis, it acts as an essential co-receptor for FGF23 to regulate phosphate excretion and vitamin D metabolism; deficiency of α-Klotho can lead to hyperphosphatemia and vascular calcification, which is highly similar to the common mineral metabolism disorders in diabetic patients (33). Secondly, α-Klotho has antioxidant and anti-inflammatory effects. It can activate Nrf2 signaling pathway, promote the expression of downstream antioxidant enzymes (such as SOD2 and NQO1), reduce oxidative stress in high glucose environment, inhibit NF-κB-mediated inflammatory response, and reduce the release of inflammatory factors (34, 35). Third, α-Klotho prevents excessive cell proliferation and tissue fibrosis by inhibiting TGF-β/Smad and Wnt/β-catenin signaling pathways in anti-fibrosis and anti-apoptosis. This effect has been confirmed in the kidney, heart and retina (24, 36, 37). Finally, α-Klotho can regulate energy metabolism and partially inhibit the insulin/IGF-1 signaling pathway, thereby delaying cell senescence and providing a theoretical basis for its potential role in the development of diabetes.

The regulation of α-Klotho expression is multifactorial and includes FGF23-mediated feedback, inflammatory cytokines, oxidative stress, metabolic dysfunction, and renal injury. Conditions such as hyperglycemia, uremia, and chronic inflammation markedly suppress Klotho transcription in the kidney, whereas exercise, calorie restriction, and certain small molecules have been reported to upregulate its expression (33, 38). Beyond the kidney, α-Klotho is also expressed in the choroid plexus, hippocampus, cerebellum, parathyroid gland, and reproductive tissues. Brain-derived Klotho has been shown to regulate synaptic function, cognitive performance, neuroinflammation, and aging-related neuronal vulnerability (39, 40). In terms of tissue distribution, the kidney is the organ with the highest expression of α-Klotho. Although the distal convoluted tubule (DCT) is the main site of synthesis, recent studies have confirmed that the proximal tubule (PCT) also expresses α-Klotho at lower levels. Importantly, many of the major biological actions of Klotho—such as regulation of phosphate handling, modulation of oxidative stress, and protection against tubular injury—primarily occur in the proximal tubule, highlighting that both nephron segments contribute to Klotho biology (41). Alpha-Klotho exists in several molecular forms, including a full-length transmembrane protein, a soluble form generated by ectodomain shedding, and a secreted truncated isoform produced by alternative splicing. The transmembrane form functions as a co-receptor for FGF23, whereas the soluble form exerts endocrine, paracrine, and autocrine actions on multiple organs. Furthermore, sKlotho attenuates IGF-1 signaling by inhibiting IGF-1R autophosphorylation, activating FOXO transcription factors, and enhancing antioxidant defenses (42). These pleiotropic actions underscore sKlotho as a multifunctional endocrine factor with broad protective effects across metabolic, renal, cardiovascular, and neural tissues. sKlotho also binds to the TGF-β type II receptor, preventing ligand–receptor interaction and inhibiting Smad2/3 phosphorylation, thereby exerting strong antifibrotic effects. It additionally inhibits canonical Wnt/β-catenin signaling by functioning as a decoy receptor for Wnt ligands, suppressing epithelial–mesenchymal transition and cellular senescence (41, 43). The mechanisms by which soluble Klotho (sKlotho) exerts systemic effects are increasingly understood. sKlotho can act as a circulating hormone, an autocrine/paracrine factor, or a molecular decoy. It interacts with multiple ion channels and transporters, including TRPV5, TRPC6, Na^+^/K^+^-ATPase, and NaPi-IIa. Alpha-Klotho contains two internal glycosidase-like domains, KL1 and KL2, which together form the extracellular portion of the protein. Although both domains share homology with family 1 β-glycosidases, they lack enzymatic activity due to mutations in catalytic residues. Emerging studies suggest that KL1 and KL2 contribute distinct biological functions. KL1 is primarily responsible for modulating ion channel activity, whereas KL2 plays a key structural role in stabilizing FGF23–FGFR complexes.

Soluble α-Klotho also interacts directly with circulating FGF23 and with multiple FGFR isoforms expressed on pancreatic β cells, vascular endothelial cells, and other metabolically active tissues (44, 45). These interactions may have important physiological implications. sKlotho binding to FGFRs in pancreatic β cells has been shown to modulate insulin secretion and protect against β−cell oxidative stress. In endothelial cells, sKlotho–FGFR engagement enhances nitric oxide production, improves endothelial function, and attenuates vascular inflammation, which may partly explain the vascular protective effects observed in diabetic models. Furthermore, sKlotho–FGFR signaling in peripheral metabolic tissues may influence glucose uptake, mitochondrial homeostasis, and cellular resilience to metabolic stress. Together, these interactions suggest that sKlotho functions not only as a co-receptor for FGF23 but also as an endocrine modulator that fine−tunes FGFR signaling pathways across multiple organs relevant to diabetes and its complications. Together, the expression of α- and β-Klotho suggests that the pancreas is both a target and an active regulator within the broader FGF–Klotho endocrine network. Their combined activity helps maintain β-cell integrity, modulate insulin dynamics, and protect the pancreas from metabolic injury, providing mechanistic insight into why Klotho deficiency contributes to impaired insulin secretion and diabetes progression. β-Klotho serves as the essential co-receptor for FGF21 in pancreatic islets, forming β-Klotho–FGFR1c complexes that mediate FGF21-driven enhancement of insulin secretion, preservation of β-cell mass, and suppression of ER-stress–induced apoptosis. The β-Klotho–FGF21 pathway also influences glucagon secretion from α cells and coordinates systemic energy balance. Both α-Klotho and β-Klotho are expressed in pancreatic islet cells, and emerging evidence indicates that their presence in the pancreas has important metabolic and endocrine implications. α-Klotho is detected predominantly in β cells and to a lesser extent in δ cells, where it contributes to the regulation of insulin secretion, protection against oxidative stress, and maintenance of mitochondrial function. Through modulation of IGF-1/FOXO signaling and attenuation of intracellular reactive oxygen species, α-Klotho supports β-cell survival under metabolic stress.

### 2.3 β-Klotho and FGF19/FGF21 pathway

Unlike α-Klotho, β-Klotho is not directly involved in anti-aging, but plays a role as an important node in metabolic regulation. It mainly acts on the liver through the FGF19-β-Klotho-FGFR4 pathway, regulating bile acid synthesis and lipid metabolism; fGF21-β-Klotho-FGFR1c pathway promotes glucose uptake, enhances insulin sensitivity and improves lipid mass spectrometry in fat and liver (19, 46).FGF21 analogues have entered clinical trials in diabetes research, showing improved effects on blood glucose and lipid metabolism, and β-Klotho is a central molecule necessary for this signal transduction (47). Therefore, β-Klotho is not only the focus of basic research, but also an important target for the development of new metabolic drugs.

### 2.4 The relationship between Klotho and diabetes

The potential mechanism of Klotho in diabetes can be summarized as ‘ three pathways ‘. First, protect target organs: Klotho alleviates the damage of high glucose environment to renal podocytes, retinal ganglion cells and cardiomyocytes through antioxidant, anti-inflammatory and anti-fibrotic effects. Secondly, regulating metabolic homeostasis: α-Klotho and β-Klotho affect insulin sensitivity, glucose metabolism and lipid balance through different signaling pathways, thus participating in the regulation of systemic energy homeostasis. Finally, in terms of genetic association, Klotho gene polymorphisms (such as G-395A, C1818T, KL-VS) are differentially associated with the risk of type 2 diabetes and its complications in different ethnic groups, providing a potential basis for individualized intervention and precision medicine (17, 20, 48, 49).In summary, Klotho is not a single functional ‘ anti-aging factor ‘, but a multifunctional protein involved in mineral metabolism, energy balance, oxidative stress and inflammation regulation. In the field of diabetes, α-Klotho and β-Klotho play two core roles in organ protection and metabolic regulation, respectively. This feature determines that Klotho may become both a clinical biomarker and a new target for treatment, and lays a theoretical foundation for further exploration of its role in diabetic complications (**Table 1**).

**Table 1:** Role of Klotho family members in diabetes.

Subtype	Expression site	Mechanism	Pathway	Role in diabetes
α-Klotho	Distal renal tubules, choroid plexus, parathyroid gland	Mineral metabolism regulation, antioxidant, anti-inflammatory, anti fibrosis, and inhibition of cell apoptosis	Nrf2、Wnt/β-catenin、TGF-β/Smad、FGF23	Protecting the kidneys/heart/retina, delaying the progression of complications (13, 14, 33)
β-Klotho	Liver, adipose tissue, pancreas	Regulation of glucose and lipid metabolism, maintenance of insulin sensitivity, and maintenance of energy balance	FGF19/FGFR4、FGF21/FGFR1c	Improving insulin resistance as a potential therapeutic target (20, 44, 45, 81)
γ-Klotho	Reproductive system, some tumor tissues	The function is not clear, and we are exploring its interaction with the FGF family	To be clarified	No directly related evidence (15, 16)
Soluble α - Klotho	blood, urine	Systemic antioxidant activity, regulation of ion channels, inhibition of IGF-1 signaling, anti fibrosis	TRPV5, Na ^+^/K ^+^ - ATPase, TGF - β R II	Can be used as a biomarker for predicting disease progression as a complication (41, 67, 69)
Klotho derived peptides	Artificial synthesis/recombination	Simulate full-length protein function and target regulated signaling pathways	Wnt/β-catenin、FGFR1c	Can be used as a candidate drug for diabetes nephropathy/retinopathy intervention (24, 29)
Truncated alpha Klotho	Renal and pancreatic beta cells	Regulating insulin secretion, protecting beta cell function, and reducing oxidative stress damage	IGF-1/FOXO、Nrf2	Improve the survival of β cells and delay the progress of diabetes (44, 45, 48)

## 3 Basic and preclinical evidence: from mechanism to organ protection

### 3.1 Podocyte injury and renal protection

**Figure 1:**
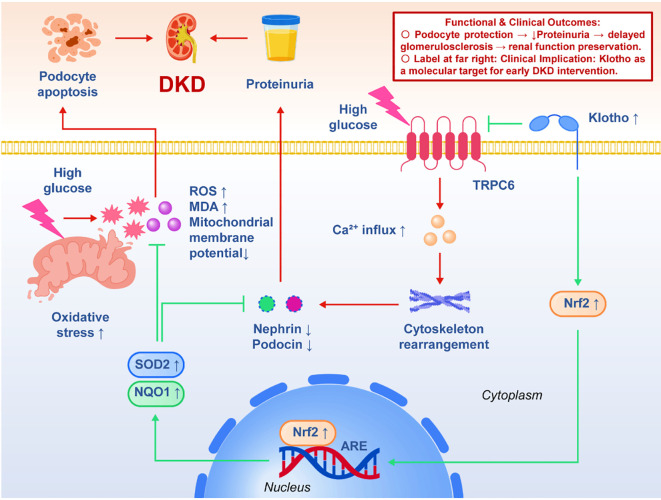
Schematic illustration of the protective mechanism of Klotho in diabetic kidney disease (DKD) podocytes.

Diabetic kidney disease (DKD) is one of the most common microvascular complications in diabetic patients and the leading cause of end-stage renal disease (ESRD). Its early pathological features are podocyte injury and glomerular filtration barrier damage. Podocytes are highly differentiated terminal cells located on the outer surface of glomerular capillaries and maintain selective filtration function by relying on the foot processes and the slit diaphragm between them (6, 50). Once the number of podocytes is reduced or the structure is damaged, it will lead to the occurrence of proteinuria and promote glomerulosclerosis and decreased renal function. Therefore, podocytes are considered to be the ‘ weak link ‘ in the progression of diabetic nephropathy (51).In this pathological context, the role of Klotho is gradually revealed. Xing et al. (2021) found that Klotho overexpression could significantly activate Nrf2 (nuclear factor erythroid 2-related factor 2) signaling pathway in high glucose-induced podocyte model *in vitro* and diabetic mice. As a classical antioxidant transcription factor, Nrf2 can enter the nucleus and bind to the antioxidant response element (ARE) to initiate the expression of downstream antioxidant genes such as SOD2 (manganese superoxide dismutase) and NQO1 (quinone reductase 1). The results showed that the level of ROS in Klotho overexpression group decreased significantly, lipid peroxidation products such as MDA decreased, and mitochondrial membrane potential was maintained. More importantly, when researchers added Nrf2 specific inhibitors, the protective effect of Klotho was partially lost and the podocyte apoptosis rate increased again, suggesting that the causal chain of Klotho → Nrf2 → antioxidant genes → podocyte protection was clearly established. Another important evidence comes from the study of Yao et al. (2022). They found that transient receptor potential channel C6 (TRPC6) was over-activated in podocytes under high glucose conditions, resulting in abnormal influx of calcium ions, causing actin skeleton rearrangement and foot process collapse, and ultimately destroying the integrity of the slit diaphragm (21).Klotho supplementation can effectively inhibit the excessive activation of TRPC6, reduce calcium influx, stabilize podocyte cytoskeleton structure, and reduce the loss of slit diaphragm proteins (such as nephrin, podocin). The further mechanism is speculated that Klotho plays a targeted protective role by regulating membrane surface receptor signaling or indirectly affecting calcium signaling pathway. This shows that Klotho is not only a global antioxidant factor, but also can accurately act on ion channel homeostasis and maintain the structural and functional integrity of podocytes at the cellular level. From the functional results, whether Xing or Yao ‘s experiments, Klotho intervention can significantly reduce the apoptosis rate of podocytes, improve urinary albumin excretion, and slow down glomerulosclerosis and renal function deterioration in animal models. These studies provide full-link evidence from molecular mechanism → cell function → animal phenotype, and strengthen the rationality of Klotho as a molecular target for early intervention in DKD. The clinical implication is that if exogenous Klotho supplementation or endogenous Klotho expression can be promoted in the early stage of diabetes, it is expected to protect podocytes before the occurrence of proteinuria, thereby delaying or even blocking the occurrence of DKD. Compared with traditional markers that can only reflect the results of injury, Klotho ‘s role is more biased towards the ‘ course-driven link ‘, which makes it particularly valuable in future precise interventions. We further constructed a schematic model illustrating the protective mechanism of Klotho in podocytes under DKD conditions (**Figure 1**).

### 3.2 Evidence in animal models

In the study of diabetic animal models, the intervention effect of exogenous supplementation of Klotho or its derivatives on renal injury has been increasingly verified. First, in the Akita mouse model, the model is considered to be one of the classic models of type 1 diabetes (T1DM) due to insufficient insulin secretion caused by Ins2 gene mutation, resulting in spontaneous hyperglycemia. Kadoya et al. (2016) showed that the kidney weight of Akita mice was significantly reduced after overexpression of Klotho in Akita mice by genetic engineering, suggesting that the early pathological change of renal hypertrophy was inhibited. At the same time, electron microscopy showed that the typical glomerular basement membrane (GBM) thickening and foot process fusion in diabetic mice were significantly improved. This shows that Klotho has a protective effect on the early structural damage of diabetic nephropathy, and may delay the progression of the disease by maintaining the integrity of podocytes (52). Secondly, in the db/db mouse model, obesity, insulin resistance and hyperglycemia occur due to leptin receptor deficiency, which is a common research object of type 2 diabetes mellitus (T2DM). Takenaka et al. (2019) observed a decrease in blood pressure in db/db mice by exogenous injection of recombinant Klotho protein, suggesting that Klotho may play a regulatory role in diabetes-related hypertension by improving renin-angiotensin system (RAAS) activity or vasomotor function. At the same time, renal histological examination showed that glomerular hypertrophy was reduced, interstitial fibrosis was reduced, and collagen deposition was reduced (53). These results suggest that Klotho can also alleviate renal pathological remodeling and fibrosis in the context of T2DM, and has a universal protective effect across diabetes types. More importantly, research on Klotho derivatives. Chen et al. (2022) proposed that Klotho-derived short peptide fragments can specifically inhibit the Wnt/β-catenin signaling pathway (24).In diabetic kidney injury, the continuous activation of the Wnt/β-catenin pathway is considered to be an important driving force for glomerular sclerosis, abnormal proliferation of mesangial cells, and fibrosis. Animal experiments showed that after administration of Klotho-derived peptides, the expression of β-catenin protein in the kidney of mice decreased, the levels of downstream fibrosis markers (such as α-SMA, collagen I) decreased significantly, and the glomerular sclerosis index and interstitial fibrosis score were improved. This result suggests that Klotho-derived peptides can not only partially simulate the function of full-length Klotho, but also have the advantages of strong molecular targeting and good stability, providing a more feasible direction for drug development. Speer and Schunk (2022) further summarized the above findings, emphasizing that the role of Klotho is not limited to being a marker after renal injury, but may be directly involved in the development of the disease by regulating core mechanisms such as Wnt signaling pathway, that is, becoming a ‘ driver ‘ rather than a ‘ bystander ‘ (54). This view elevates the research of Klotho from “ passive association “ to “ core link of pathological mechanism, “ and also means that if effective Klotho derivatives or up-regulation can be developed, it may fundamentally interfere with the occurrence and development of diabetic nephropathy. In general, animal model studies have depicted a relatively complete causal chain for us: high glucose environment/insulin resistance → endogenous Klotho down-regulation → oxidative stress, inflammation and abnormal activation of Wnt/β-catenin pathway → podocyte injury, glomerulosclerosis, interstitial fibrosis → DKD progression; the intervention of exogenous Klotho or Klotho derived peptide can block this chain in different links, so as to delay or reverse the effect of DKD. These preclinical evidences provide a solid theoretical basis for the clinical transformation of Klotho in diabetic nephropathy, and suggest that future research can promote the development of related drugs from different perspectives such as gene therapy, recombinant proteins, derived peptides, and even small molecule agonists.

### 3.3 Retinal protective effect

**Figure 2:**
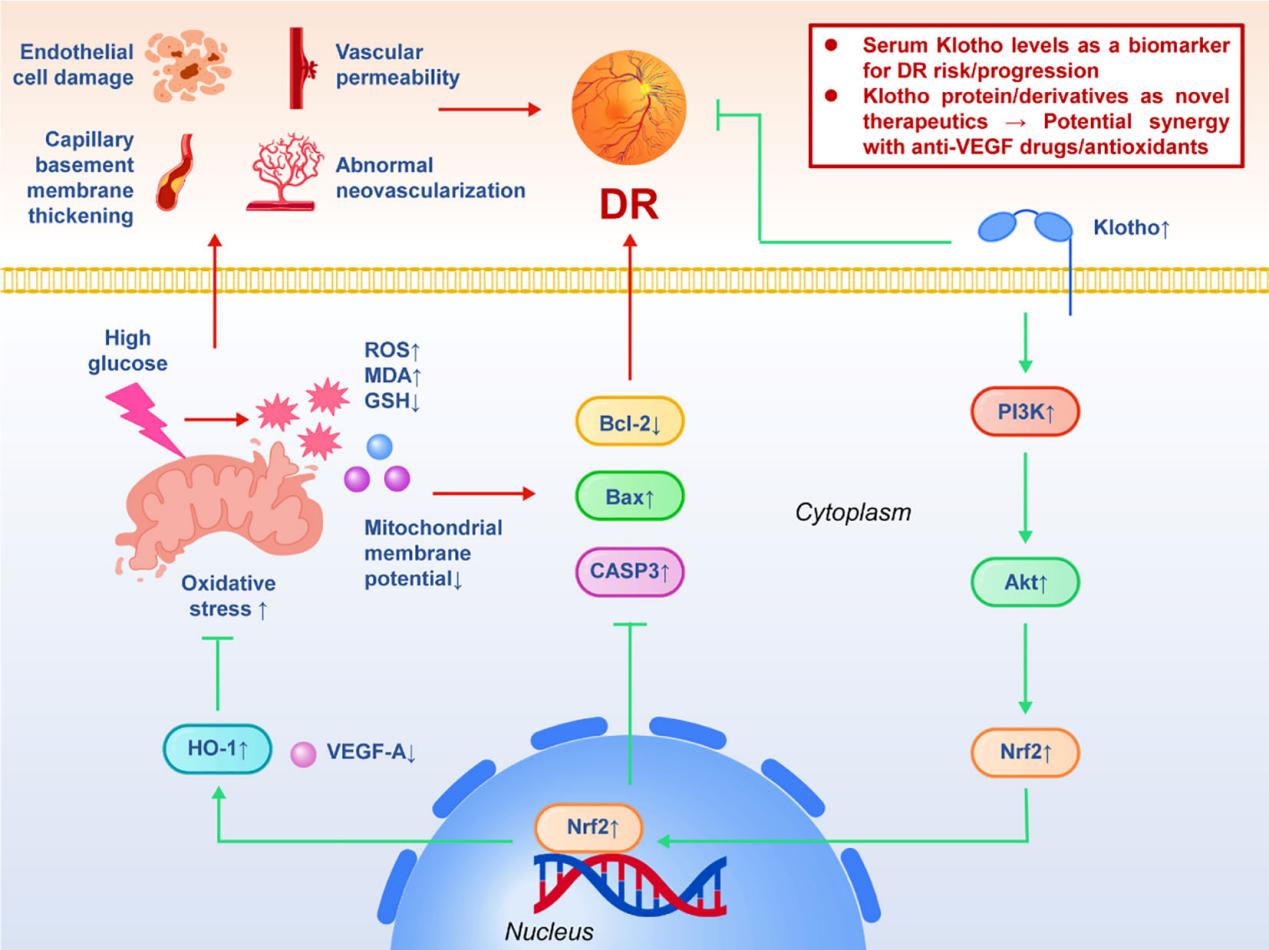
The protective mechanism of Klotho in diabetic retinopathy (DR).

Diabetic retinopathy (DR) is one of the most common microvascular complications of diabetes and the main cause of blindness in adults. According to epidemiological statistics, about one-third of diabetic patients will have varying degrees of DR, and about one-third of them will progress to severe DR or macular edema that threatens vision (55, 56). Its pathological process mainly includes high glucose-induced microvascular endothelial injury, capillary basement membrane thickening, increased vascular permeability, abnormal neovascularization, and ultimately lead to retinal ischemia, hemorrhage and fiber hyperplasia. Oxidative stress, inflammation and apoptosis are thought to be the key mechanisms driving this process. In this context, Klotho has entered the field of vision of researchers as an anti-aging molecule. Ji et al. (2020) detected serum Klotho levels in patients with different stages of diabetes in a clinical study. The results showed that compared with healthy controls, serum Klotho levels in diabetic patients, especially those with DR, decreased significantly, and the degree of decline was negatively correlated with the severity of retinopathy. *In vitro* experiments further demonstrated that Klotho expression was also down-regulated in high glucose-stimulated retinal cells (including retinal ganglion cells and vascular endothelial cells), suggesting that high glucose environment may directly inhibit Klotho transcription or translation (57). Functional experiments provide causal evidence. By adding recombinant Klotho protein, the researchers found that the levels of ROS and malondialdehyde (MDA) in retinal cells damaged by high glucose were significantly decreased, the level of glutathione (GSH) was restored, and the mitochondrial membrane potential was partially maintained. At the same time, TUNEL staining showed that Klotho could reduce the number of apoptotic cells under high glucose conditions. Western blot results showed that Bcl-2 was up-regulated, Bax was down-regulated, and caspase-3 cleavage was reduced, which proved that Klotho protected cell survival through a mitochondrial-dependent apoptotic pathway. Further mechanism research came from Wen et al. (2022). In an *in vitro* model of human retinal pigment epithelial cells (RPE), they used hydrogen peroxide (H 2–2 O 2) to simulate oxidative stress injury. The results showed that after treatment with recombinant human Klotho protein, the cell survival rate was significantly increased, the ROS accumulation was reduced, and the antioxidant enzyme activity was enhanced. Molecularly, Klotho significantly activated the PI3K/Akt pathway and further up-regulated the nuclear translocation of the transcription factor Nrf2 and the expression of the downstream antioxidant protein HO-1. This protective effect was significantly attenuated after the use of PI3K inhibitors, suggesting that the PI3K/Akt-Nrf2/HO-1 axis plays a key role in the antioxidant effect of Klotho (22, 58). In addition, other studies have suggested that Klotho may also play an anti-vascular pathological role in proliferative DR by inhibiting the excessive secretion of VEGF-A and reducing the abnormal proliferation of new blood vessels. This echoes its anti-angiogenic effect in kidney disease models, suggesting that Klotho may play a general protective role in multiple organ microangiopathy. These experimental results together depict a relatively complete causal chain: high glucose environment/oxidative stress → Klotho down-regulation → ROS accumulation and mitochondrial damage → apoptosis and vascular abnormalities → DR progression; supplementing Klotho or increasing its expression can reverse ROS accumulation, restore antioxidant system, inhibit apoptosis and vascular abnormalities, thus delaying or even blocking the occurrence and development of DR. In terms of clinical implications, Klotho may have dual potential of biomarkers and intervention targets. Serum Klotho level is expected to be a predictor of risk stratification and progression of DR, and Klotho protein or derived peptide may develop into a new treatment strategy, especially in combination with anti-VEGF drugs or antioxidants, which may have a synergistic effect. A schematic model was developed to illustrate the regulatory mechanism of Klotho in diabetic retinopathy (DR) (**Figure 2**). The challenge in the future is how to achieve stable exogenous supplementation or endogenous activation of Klotho in the clinical environment and explore its binding mode with existing standard treatments.

### 3.4 Myocardial protection

**Figure 3:**
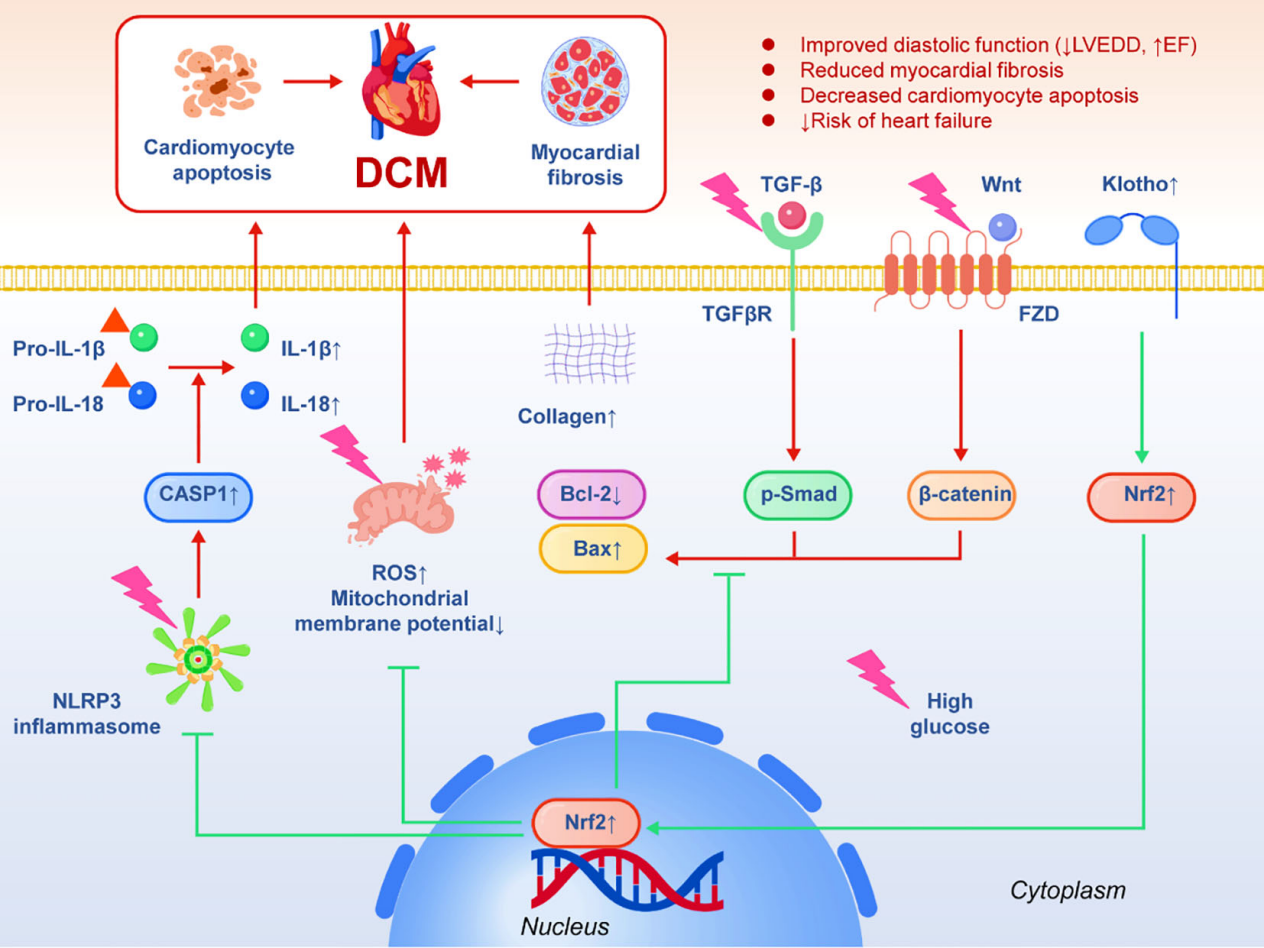
The protective mechanism of Klotho in diabetic cardiomyopathy (DCM).

Diabetic cardiomyopathy (DCM) refers to the abnormal myocardial structure and function in diabetic patients without obvious coronary heart disease, hypertension or other heart diseases such as valvular disease. Its typical manifestations include ventricular diastolic dysfunction, myocardial fibrosis and eventual heart failure (59–61). Epidemiological data show that the risk of heart failure in T2 DM patients is about 2–5 times higher than that in non-diabetic people, suggesting the direct damage of high glucose environment to myocardium. Its pathological mechanism is complex, but oxidative stress, mitochondrial dysfunction and inflammatory response are considered to be the core driving factors. In the previous clinical study, Li et al. (2019) systematically evaluated the expression of Klotho in myocardial tissue and its intervention effect using a streptozotocin (STZ) -induced diabetic rat model. The results showed that the levels of α-Klotho protein and mRNA in myocardial tissue of diabetic model rats were significantly down-regulated, accompanied by increased levels of myocardial fibrosis markers (collagen I, collagen III) and inflammatory factors (IL-1β, IL-18). Histological examination showed obvious cardiomyocyte apoptosis and interstitial fibrosis. Further mechanism studies have shown that NLRP3 inflammasome (NLR family pyrin domain containing 3 inflammasome) is highly activated in cardiomyocytes under diabetic conditions, leading to caspase-1 activation and secretion of downstream inflammatory cytokines IL-1β and IL-18, aggravating myocardial injury. After the intervention of exogenous recombinant Klotho protein, the activation level of NLRP3 inflammasome was significantly reduced, the cleavage of caspase-1 was reduced, and the release of IL-1β and IL-18 was inhibited. At the same time, Klotho can also restore mitochondrial membrane potential and reduce ROS levels, thereby reducing oxidative stress load. At the functional level, the cardiac diastolic function of the rats in the Klotho intervention group was significantly improved, and the echocardiography showed that the left ventricular end-diastolic diameter was reduced and the ejection fraction was increased. The degree of myocardial fibrosis was reduced, and TUNEL staining showed that the apoptosis rate of myocardial cells was decreased. These results together confirm the multiple protective effects of Klotho in diabetic myocardial injury. From the perspective of molecular pathways, the cardioprotective effect of Klotho on diabetic cardiomyopathy (DCM) may be achieved through multiple mechanisms. First, Klotho can inhibit the activation of NLRP3 inflammasome, reduce the release of pro-inflammatory factors and block inflammatory cell death. Secondly, it activates the Nrf2 signaling pathway, up-regulates the expression of antioxidant enzymes, improves oxidative stress and mitochondrial function, and reduces the accumulation of reactive oxygen species (ROS); at the same time, Klotho can also reduce myocardial fibrosis and collagen deposition by inhibiting TGF-β/Smad and Wnt/β-catenin signaling pathways. In addition, it inhibits cardiomyocyte apoptosis by regulating the Bcl-2/Bax ratio (62, 63). The above studies not only expand our understanding of the pathogenesis of DCM, but also suggest that Klotho may become a new target for the intervention of cardiovascular complications. If stable and effective up-regulation can be achieved clinically through gene therapy, recombinant protein or Klotho-derived peptides in the future, it is expected to provide a new treatment strategy for the prevention of heart failure in diabetic patients. A schematic model was established to elucidate the cardioprotective role of Klotho in DCM (**Figure 3**).

Basic and preclinical studies have provided sufficient evidence for the role of Klotho in diabetic complications. Its protective effect is not only reflected in the global antioxidant and anti-inflammatory levels, but also involves Nrf2, TRPC6, Wnt/β-catenin, PI3K/Akt-Nrf2/HO-1, NLRP3 and other key pathways, forming a relatively complete ‘ causal chain ‘. These results provide a solid scientific basis for Klotho as a potential therapeutic target, and also suggest that future clinical transformation can focus on Klotho derivatives and pathway-specific regulation.

## 4 Clinical and epidemiological evidence

### 4.1 The relationship between Klotho and diabetes and metabolic control

In recent years, with the increasing research on Klotho in aging and metabolic diseases, clinical epidemiological evidence has gradually focused on the correlation between serum or urine soluble Klotho (sKlotho) levels and the risk of diabetes and metabolic control. However, the results did not form a consistent conclusion, and there were significant differences between different research reports. In a cross-sectional study based on the National Health and Nutrition Examination Survey (NHANES), Wang et al. (2022) included thousands of American adults over the age of 40, and used multivariate regression models to analyze the relationship between sKlotho level and diabetes prevalence. The results showed that higher sKlotho levels were positively correlated with the prevalence of type 2 diabetes mellitus (T2DM), a finding that is counterintuitive given the traditional view of Klotho as a protective factor (64). The researchers speculate that this “positive correlation” is not a causal pathogenic relationship, but more likely to reflect the body’s compensatory response to high glucose and inflammatory load: that is, in early or preclinical diabetes, transient stress-induced upregulation of Klotho may occur to counteract oxidative stress or metabolic injury. Therefore, this phenomenon suggests that in cross-sectional population studies, sKlotho levels may be influenced by dynamic metabolic stress rather than reflecting baseline physiological status. In contrast, Ciardullo and Perseghin (2022) found in a clinical study of patients diagnosed with T2DM that lower sKlotho levels are often accompanied by poor glycemic control (higher HbA1c) and more severe renal impairment (lower eGFR). After multivariate correction, sKlotho was independently and negatively correlated with HbA1c (65). These findings imply that the decrease of sKlotho level may be closer to the phenotypic characteristics of disease progression, reflecting both cumulative metabolic burden and renal dysfunction. In this context, sKlotho may function more as an integrated indicator of long-term disease burden rather than an early diagnostic biomarker. In addition, van Ark et al. (2013) found that there was no significant difference in circulating sKlotho levels between T2DM patients without nephropathy and healthy controls in a small sample study. This result suggests that sKlotho may remain relatively stable in early diabetes or in individuals with preserved renal function, indirectly highlighting the influence of kidney status on sKlotho levels (66).

**Figure 4:**
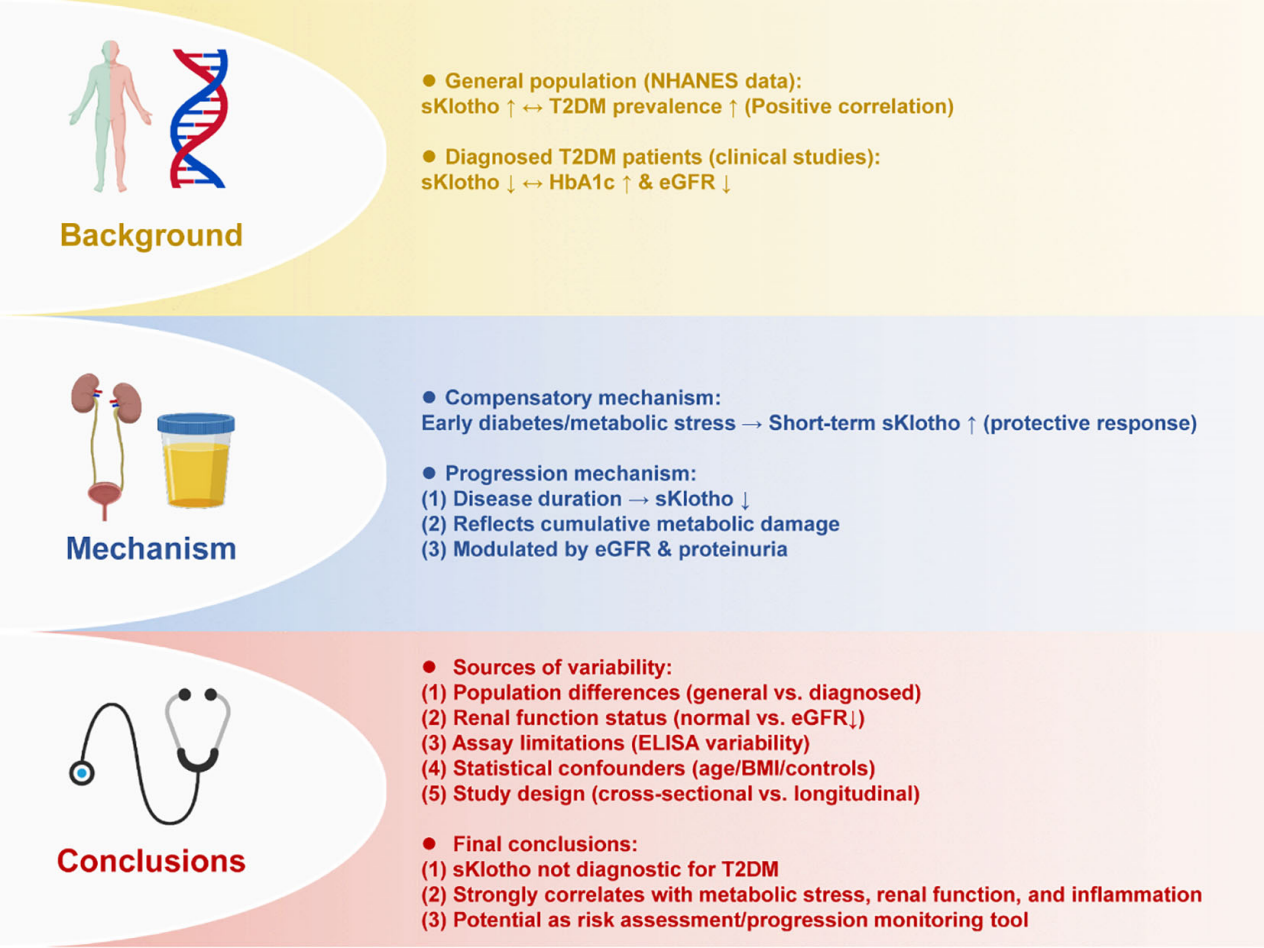
The relationship between Klotho and diabetes development and metabolic control.

The inconsistencies among different studies may arise from multiple interacting factors. First, the characteristics of the populations examined vary substantially, with general population–based datasets such as NHANES reflecting disease risk, whereas hospital-based cohorts represent more advanced disease stages and complications. Renal function serves as a major confounder, as Klotho is synthesized and partially cleared by the kidney; thus, reductions in eGFR can directly lower circulating sKlotho and contribute to discrepancies across cohorts with distinct renal profiles. Furthermore, compensatory biological responses may play a role: early hyperglycemia, inflammation, or oxidative stress can transiently elevate sKlotho, while chronic metabolic injuries suppress its expression. Ethnic, genetic, and inflammatory heterogeneity may additionally modify baseline Klotho levels. Emerging evidence also suggests that the association between Klotho and metabolic risk may follow a non-linear or U-shaped pattern, in which both very low and unusually high concentrations reflect metabolic stress. Methodological variation, including differences in ELISA sensitivity and specificity across manufacturers, inadequate adjustment for confounders (age, BMI, inflammatory markers, renal function), and heterogeneity in sample size and study design (cross-sectional versus longitudinal), also contribute to inconsistent findings. Taken together, these factors indicate that sKlotho should be interpreted in the context of disease stage, renal function, and inflammatory status, rather than as a simple high-versus-low biomarker. Clarifying whether sKlotho changes represent causal mechanisms, compensatory responses, or markers of accumulated disease burden will require standardized assays, renal function–stratified analyses, and prospective longitudinal studies. Although current evidence is insufficient to support sKlotho as a diagnostic marker for diabetes, its close relationship with metabolic stress, renal status, and systemic inflammation suggests its potential utility for disease risk assessment and progression monitoring. Future research should further delineate the temporal trajectory of sKlotho alterations during the course of diabetes. A schematic model was developed to elucidate the complex relationship between serum Klotho (sKlotho) levels, diabetes onset, and metabolic regulation (**Figure 4**).

### 4.2 Klotho as a complication marker and prognostic factor

In clinical studies of diabetic complications, Klotho has the most solid evidence, especially in diabetic kidney disease (DKD). Lee et al. (2014) first reported that Klotho levels in urine and serum decreased significantly in the early stage of renal injury in patients with T2 DM, suggesting that Klotho can be used as a sensitive early biomarker to reflect renal lesions earlier than traditional proteinuria detection (67). Further studies have found that Kacso et al. (2012) observed a correlation between sKlotho and vascular endothelial growth factor A (VEGF-A), suggesting that it may affect glomerular pathological changes by regulating angiogenesis and endothelial function (68) Follow-up studies also provide evidence of prognostic value. For example, Kim et al. (2016) reported that low levels of sKlotho can independently predict the progression of nephropathy and aggravation of proteinuria in patients with T2DM, even after adjusting for traditional risk factors (69). The results of systematic review and meta-analysis further strengthened this view: Xin et al. (2022) and Yu et al. (2023) combined a number of studies to confirm that low sKlotho was significantly associated with the occurrence and progression of DKD, supporting its application potential in clinical risk stratification (70). However, different studies have differences in detection methods, reference thresholds and population characteristics. In the future, it is urgent to establish a unified detection standard and reference range. In terms of diabetic retinopathy (DR), clinical evidence is gradually accumulating. A prospective follow-up study by Corcillo et al. (2020) found that low levels of sKlotho can predict the occurrence and progression of DR, suggesting that it is expected to be a potential indicator for early identification of fundus lesions (71). Ji et al. (2020) further confirmed this conclusion by combining clinical patient samples and *in vitro* experiments, and revealed that low Klotho levels may promote the occurrence of DR by exacerbating oxidative stress (57) However, Oner et al. (2024) found no significant correlation in non-proliferative DR patients with type 1 diabetes mellitus (T1DM), indicating that the type of disease, the stage of disease and the severity of complications may affect the relationship between Klotho and DR (72). Therefore, Klotho may be more suitable for risk prediction in the progressive or proliferative stage of DR, and its diagnostic value in early non-proliferative DR needs more research to verify. In terms of cardiovascular complications, Klotho also showed some predictive value. Cardiovascular disease is the leading cause of death in diabetic patients, and early identification of high-risk populations is crucial. Pan et al. (2018) found that low levels of sKlotho can independently predict the occurrence of macrovascular events (such as myocardial infarction and stroke) in the follow-up of patients with T2DM, suggesting that it can be used as a new tool for clinical risk assessment (73). The study of Castelblanco et al. (2022) further pointed out that in patients with T1 DM, sKlotho level was significantly correlated with carotid intima-media thickness (cIMT) and subclinical atherosclerosis, suggesting that Klotho was closely related to vascular structural remodeling (74). Bi et al. (2024) also proposed that the combined detection of FGF23 and Klotho levels can better predict the risk of atherosclerosis in patients with T2DM than the detection of Klotho alone, suggesting that there may be a synergistic or complementary effect between the two (75). These studies not only show the clinical predictive value of Klotho levels, but also suggest that it may be directly involved in the occurrence and development of cardiovascular complications through mechanisms such as anti-inflammatory, anti-calcification, and regulation of vascular endothelial function, not just an indirect reflection of renal function damage. In general, Klotho has shown its potential as a biomarker and prognostic factor in DKD, DR and cardiovascular complications. Among them, DKD has the most sufficient evidence. Although there is a certain heterogeneity in the results of DR and cardiovascular diseases, the overall trend supports its protective effect. Future research should be further optimized in the classification of the population, the stage of the disease and the standardization of detection methods, so as to lay a foundation for the transformation and application of Klotho in clinical practice.

**Figure 5:**
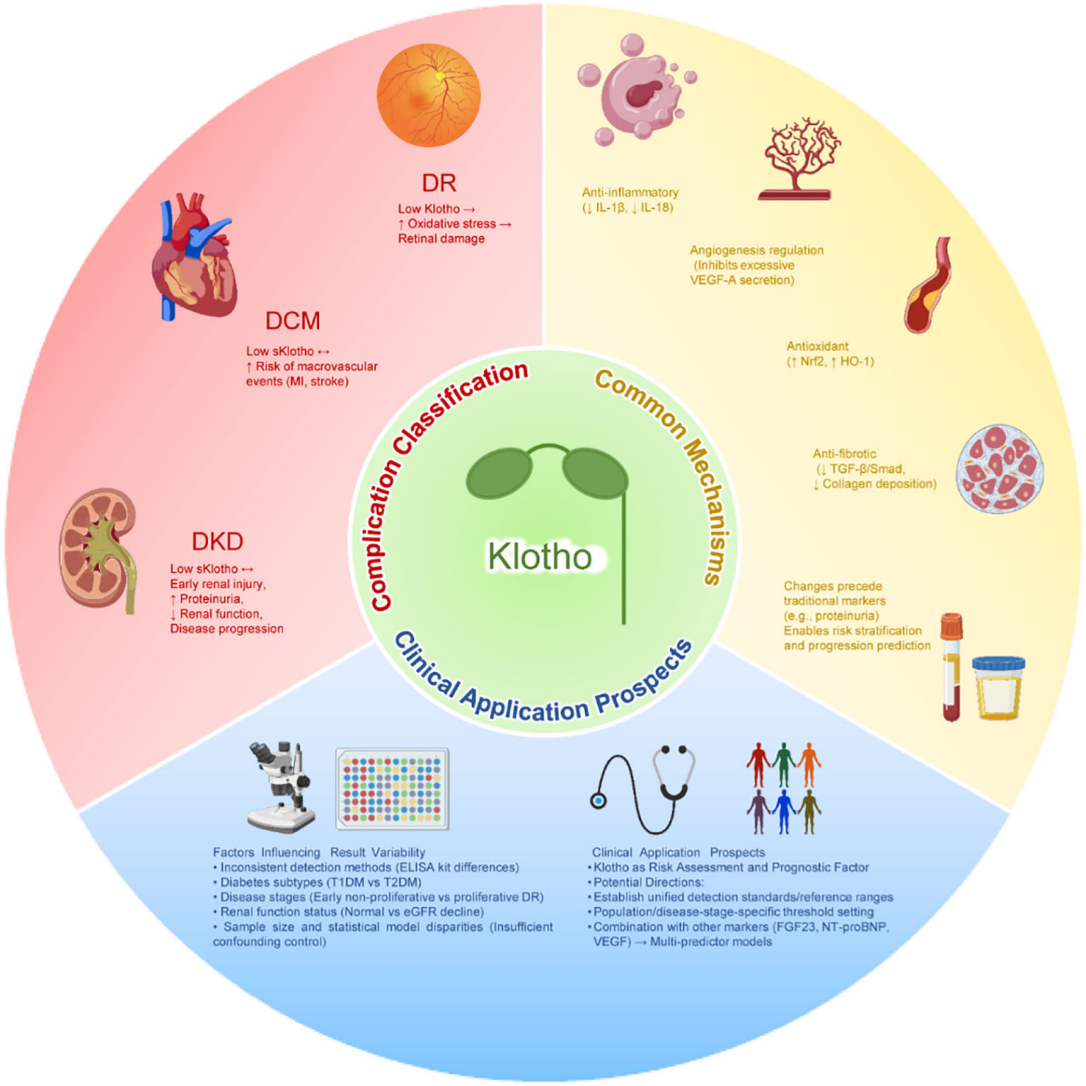
Klotho as a biomarker and prognostic factor for diabetic complications.

The overall evidence of clinical and epidemiological studies shows that Klotho is closely related to diabetes and its complications, but there are some differences between different research results. In terms of the risk of diabetes and metabolic control, the conclusions of cross-sectional and cohort studies are not consistent: some large sample population studies suggest that higher sKlotho levels are positively correlated with the prevalence of T2DM, which may reflect the compensatory up-regulation in the disease state; clinical studies on confirmed patients have generally found that lower sKlotho is closely related to poor blood glucose control and renal dysfunction. Overall, these differences more reflect the impact of renal function status and metabolic stress levels on Klotho levels. In contrast, the evidence is relatively consistent in the prediction of diabetic complications: especially in diabetic kidney disease (DKD), a number of cross-sectional studies, prospective follow-ups, and meta-analyses have confirmed that low levels of sKlotho are closely related to early renal injury, disease progression, and aggravation of proteinuria, showing its potential clinical value as a risk stratification tool. For the study of diabetic retinopathy (DR) and cardiovascular complications, although some results are heterogeneous, the overall trend still supports the protective effect of Klotho. For example, in DR patients, low Klotho is associated with lesion progression. In the cardiovascular field, low Klotho can also independently predict macrovascular events or is associated with the degree of atherosclerosis. The differences between the results of different studies are mainly due to the sample size, different detection methods, different types of diabetes and different stages of disease, and insufficient correction of confounding factors. Based on this, future research should focus on several key directions: first, establish a standardized Klotho detection system and reference range to improve the comparability between different studies; second, clarify the threshold definition of different populations and disease stages, so as to achieve more accurate hierarchical management; thirdly, to explore the value of combined application of Klotho and other markers (such as FGF23, NT-proBNP, VEGF, etc.), in order to form a multi-index combined prediction model and improve the accuracy of risk assessment and prognosis of diabetic complications. A schematic model was constructed to integrate evidence on Klotho as a biomarker and prognostic indicator for diabetic complications (**Figure 5**) (**Table 2**).

**Table 2:** Mechanism and clinical value of Klotho in complications of diabetes.

Complication	Mechanism	Pathway	Source of evidence	Clinical value
Diabetic nephropathy	Inhibit podocyte apoptosis, stabilize glomerular filtration barrier, and reduce renal fibrosis	Nrf2、TRPC6、Wnt/β-catenin、TGF-β/Smad	Multi center meta-analysis	Serum and urine levels can predict early kidney injury (21, 52, 70)
Diabetes retinopathy	Reduce oxidative stress damage, inhibit abnormal neovascularization, and protect retinal nerve cells	PI3K/Akt-Nrf2/HO-1、VEGF-A	Queue study+*in vitro* experiment	Predicting lesion progression (57, 71, 72)
Diabetes cardiomyopathy	Inhibit myocarditis, reduce myocardial fibrosis, and improve mitochondrial function	NLRP3 inflammasome、Nrf2、Wnt/β-catenin	Animal experiments+small sample clinical trials	Negative correlation with cardiac function indicators, which can serve as potential predictive indicators (62, 63)
Diabetes cardiovascular disease	Anti vascular calcification, improving endothelial function, inhibiting atherosclerosis	FGFRs、NF-κB	Queue research+mechanism verification	Can serve as potential predictive indicators for myocardial infarction and stroke (73–75)
Neuropathy in diabetes	Reduce oxidative stress in nerve cells and inhibit the release of inflammatory factors	Nrf2、NF-κB	A small number of animal experiments	Potential predictive indicators but insufficient evidence at present (16, 26, 34, 35)
Metabolic syndrome	Improving insulin resistance and regulating glucose and lipid metabolism balance	FGF21/β-Klotho、IGF-1	cross-sectional study	Expression level is negatively correlated with disease risk (49, 82, 83)

## 5 Genetic polymorphism and individualized medicine

**Figure 6:**
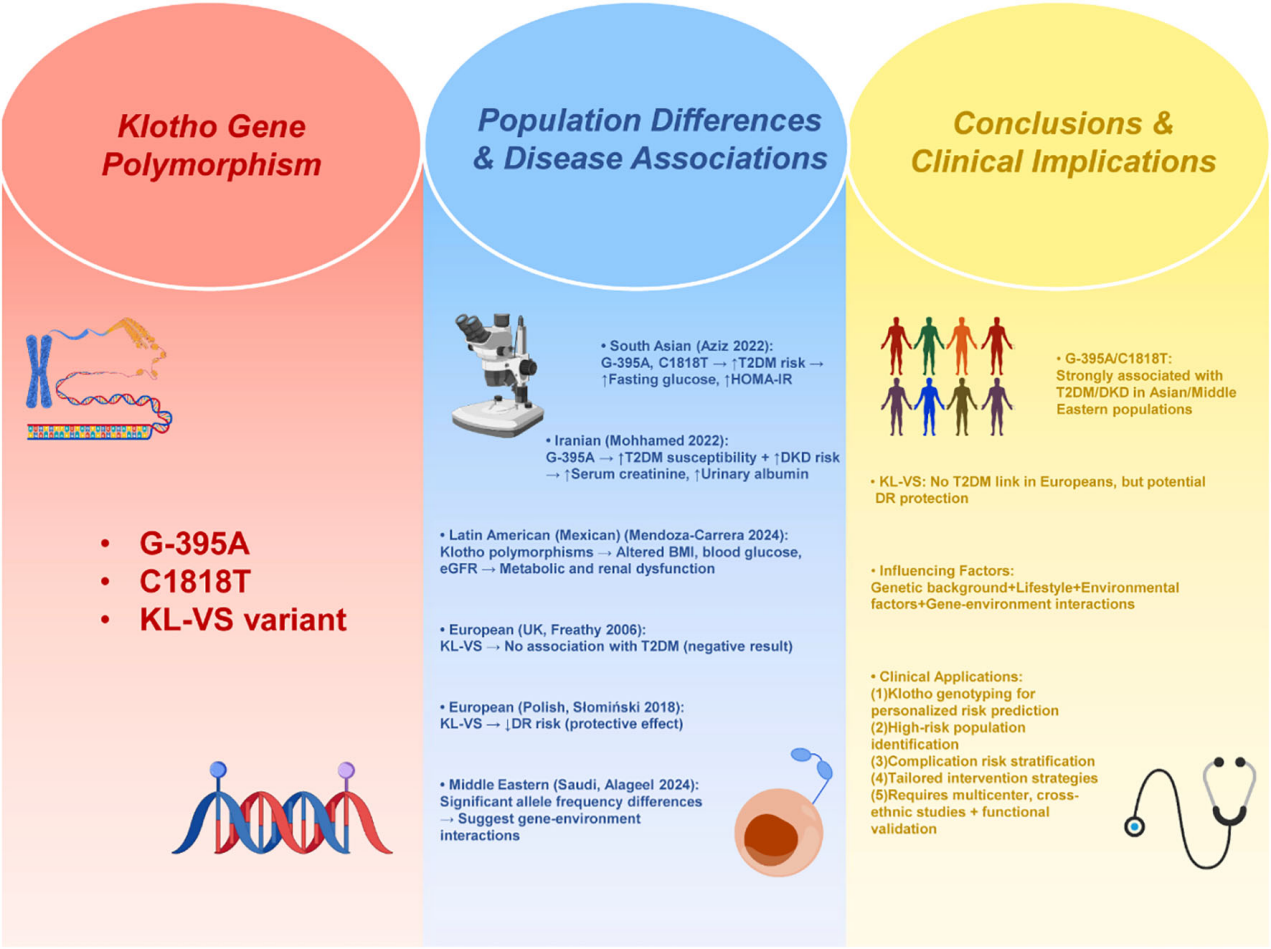
Schematic illustration of Klotho gene polymorphisms and their associations with diabetes and its complications.

In recent years, with the continuous development of genomics and individualized medicine, researchers have gradually focused on the role of Klotho gene polymorphism in diabetes and its complications. However, the current research results are not completely consistent, and there are great differences among different ethnic groups and different disease types. Among the common single nucleotide polymorphisms (SNPs), G-395A and C1818T have received the most attention. Aziz et al. (2022) reported in the South Asian population that the mutant alleles of these two loci were significantly associated with an increased risk of T2 DM, and were positively correlated with elevated fasting blood glucose and deterioration of insulin resistance index (HOMA-IR) (76). Mohhamed et al. (2022) further found in the Iranian population that the G-395A variant was not only associated with the susceptibility to T2DM, but also with the increased risk of diabetic kidney disease (DKD), and was significantly associated with clinical indicators such as elevated serum creatinine and increased urinary albumin excretion, suggesting that these sites may play a role in disease susceptibility and risk prediction of complications (76). In Latin American population, Mendoza-Carrera et al. (2024) found that the polymorphism of Klotho gene is closely related to metabolic phenotype (such as BMI, blood glucose level) and renal function parameters (such as eGFR) in Mexican patients with T2DM, suggesting that the genetic effect of Klotho not only affects the risk of diabetes, but also may change the disease process by regulating metabolism and renal function (77). In contrast, large sample studies in European populations have reached different conclusions: Freathy et al. (2006) analyzed functional KL-VS variants in more than 5,000 white British samples and found no significant association with T2DM risk, suggesting that KL-VS is not a universally applicable genetic risk locus (78);However, in terms of complications, Słomiński et al. (2018) found a negative correlation between KL-VS variants and the risk of diabetic retinopathy (DR) in the Polish T1DM cohort, that is, a protective effect, indicating that it may play a role in the occurrence of microvascular complications rather than diabetes itself (79). At the same time, Alageel et al. (2024) emphasized the importance of ethnic differences in the Saudi population, and found that the allele frequency of the Klotho gene was significantly different between different ethnic groups. For example, it showed a strong association in the Asian population. Mutations may have no effect in the European population, further indicating that the Klotho polymorphism effect may depend on genetic background, lifestyle, environmental factors and gene-environment interactions (80). In general, existing evidence suggests that Klotho gene polymorphisms may be valuable in predicting the risk of specific populations and specific complications. For example, G-395A and C1818T are closely related to T2DM and DKD in Asian and Middle Eastern populations, while KL-VS may have a protective effect on microvascular complications (such as DR) in European populations. A schematic overview of Klotho gene polymorphisms and their clinical implications was developed to explore the genetic contribution of Klotho variants to diabetes susceptibility and complications (**Figure 6**). These findings not only suggest the potential application value of Klotho genotype detection in individualized medicine and precise intervention of diabetes in the future, but also indicate that it may be used to identify high-risk groups, predict the risk of complications, and guide the formulation of intervention strategies. However, due to the significant differences between different ethnic groups, more large-scale, multi-center, cross-ethnic studies are still needed, combined with functional experiments to further clarify the real biological significance and clinical transformation potential of these gene mutations.

## 6 The therapeutic implications of β-Klotho/FGF19/FGF21 axis

In the study of diabetes, in addition to the widely concerned α-Klotho, β-Klotho has also been paid more and more attention in recent years. β-Klotho is mainly highly expressed in liver, adipose tissue and pancreas, and is an indispensable core co-receptor for FGF19 and FGF21 signaling pathways (20). It can form a complex with FGFR1c, FGFR3c, FGFR4 and other receptors, so that FGF19/21 can play a tissue-specific metabolic regulation role. Specifically, FGF19 inhibits bile acid synthesis, promotes hepatic glycogen synthesis, regulates lipid metabolism, and improves overall energy balance through the β-Klotho-FGFR4 pathway. FGF21 enhances insulin sensitivity, promotes fatty acid oxidation and blood glucose decline in adipose tissue, liver and islet β cells through the β-Klotho-FGFR1 c pathway, while inhibiting liver gluconeogenesis and participating in the regulation of systemic metabolic homeostasis (81). It is worth noting that the loss or down-regulation of β-Klotho expression can significantly weaken the metabolic effect of FGF21, which may explain the phenomenon of ‘ tolerance ‘ or poor efficacy of FGF21 drugs (such as LY2405319, pegbelfermin, PF-05231023, etc.) in some patients. Animal model studies have shown that in β-Klotho deficient mice, the glucose and lipid regulation of FGF21 is significantly weakened, and its metabolic effects can be partially restored by gene knock-in or recombinant protein supplementation, which provides an experimental basis for the therapeutic potential of β-Klotho. In addition, β-Klotho activation may also affect the occurrence and progression of diabetic complications, such as reducing cholesterol synthesis and fat deposition in the liver and improving non-alcoholic fatty liver disease. It can improve insulin resistance-related vascular dysfunction in the cardiovascular system; in the kidney, it may reduce the early damage of diabetic nephropathy by regulating metabolic and anti-inflammatory pathways. In recent years, Hua et al. (2021) pointed out that β-Klotho itself is not only an ‘ essential switch ‘ of FGF19/21 signal, but also may become an independent therapeutic target: increasing the expression of β-Klotho through small molecule drugs or gene regulation can not only enhance the efficacy of FGF21 drugs, but also directly improve insulin resistance, blood glucose and lipid metabolism abnormalities (20). In disease models such as metabolic syndrome, nonalcoholic fatty liver disease, and obesity, β-Klotho activation has shown the potential to improve fatty acid oxidation, reduce inflammation levels, regulate liver cholesterol synthesis, improve islet β-cell function, and reduce islet fibrosis. In addition, β-Klotho and α-Klotho may have synergistic or complementary effects in the regulation of metabolic homeostasis: α-Klotho protects the kidney and cardiovascular system mainly by regulating insulin/IGF-1 signaling, anti-oxidation and anti-fibrosis, while β-Klotho enhances FGF19/21 signaling in liver and adipose tissue to achieve systemic metabolic regulation. The combined analysis of the two may provide a more accurate tool for diabetes risk assessment and complication prediction. Future studies should further explore the expression differences of β-Klotho in different populations, ages, obesity degrees and metabolic states, and clarify the value of combined detection of β-Klotho, α-Klotho, FGF23 and other molecules in the risk prediction of diabetes and metabolic syndrome. At the same time, β-Klotho agonists or combined strategies with FGF21 drugs should be explored to systematically study dose dependence, tissue specificity and long-term safety. In terms of molecular mechanisms, β-Klotho may have complex interactions with inflammatory signals (such as NF-κB), oxidative stress pathways (such as Nrf2), lipid metabolism regulators (such as SREBP1 c, PPARα), and islet cell autophagy and ER stress pathways. In the future, in-depth study of its network effects will help to reveal the multiple roles of β-Klotho in systemic metabolic homeostasis and the course of diabetes, and provide theoretical and experimental basis for precise intervention.

Although Klotho-based therapies demonstrate promising efficacy in preclinical models, several safety concerns must be addressed before clinical translation. Potential off-target effects may arise because Klotho is involved in multiple signaling pathways, including insulin/IGF-1, Wnt/β-catenin, and mineral metabolism, raising the possibility of unintended metabolic disturbances. In addition, recombinant Klotho proteins or Klotho-derived peptides may induce immunogenic reactions during repeated or long-term administration. Another challenge is the risk associated with chronic or excessive Klotho overexpression, which could disrupt phosphate homeostasis, alter FGF23/FGF21 signaling balance, or exaggerate anti-fibrotic effects, potentially impairing normal tissue repair. Moreover, the long-term systemic consequences of Klotho augmentation remain unclear due to the lack of toxicology and dose-response data in humans. Therefore, future studies should incorporate comprehensive safety evaluation, including pharmacokinetics, immunotoxicity, and long-term metabolic monitoring, to ensure that therapeutic benefits are balanced with potential risks (**Table 3**).

**Table 3:** Advantages and disadvantages of Klotho related treatment strategies.

Treatment method	Target site	Advantage	Limitation
Recombinant α - Klotho protein	Whole body multi-target (Nrf2, TGF - β R II, etc.)	Comprehensive function and protection of multiple organs	Short half-life, high frequency of administration, high production cost (52, 53)
Klotho derived peptides	Wnt/β - catenin, TRPV5, TGF - β R II	Strong targeting, high stability, and minimal side effects	Narrow scope of action, requiring combination therapy (24, 29)
β - Klotho agonist	β - Klotho/FGFR1c complex	Enhance the efficacy of FGF21 and improve insulin resistance	Insufficient tissue specificity may affect bile acid metabolism (20, 47)
FGF21 analog combined with β - Klotho	β-Klotho/FGFR1c	It has entered clinical practice and the effect of blood glucose regulation is clear	Some patients have tolerance and depend on β - Klotho expression levels (19, 46, 81)
Gene therapy promotes Klotho overexpression	Kidney/pancreas targeted gene delivery	Long term expression, long-term benefits from a single administration	Security to be verified, delivery system efficiency limited (44, 45, 54)
Small molecule compounds upregulate endogenous Klotho	Klotho transcription regulatory factors (such as Nrf2 agonists)	Oral administration, convenient use, low cost	Limited regulatory efficiency and significant individual response differences (34, 35)
FGF23 inhibitor	FGF23/FGFR complex	Improve mineral metabolism disorders and synergize Klotho effects	May affect calcium phosphorus balance and require strict monitoring (33, 38–40)

## 7 Methodology and transformation challenges

Although more and more basic and clinical studies have suggested that Klotho has important value as a potential biomarker and intervention target for diabetes and its complications, it still faces multiple challenges in methodology and clinical transformation. First, the detection method has not been standardized. At present, commercial ELISA kits are mostly used to determine serum or urine Klotho levels, but the sensitivity and specificity of different manufacturers and different batches are significantly different. At the same time, the sample collection time (fasting/non-fasting), storage temperature, freeze-thaw times and anticoagulant type may affect the determination results, which makes it difficult to directly compare the data between studies (82, 83). In the future, it is necessary to establish a unified detection standard, including sample pretreatment process, quality control system and calibration curve, so as to ensure the consistency and repeatability of cross-center research. Secondly, the confounding effect of renal function is difficult to avoid. Klotho is mainly synthesized and secreted in the kidney, and its serum level is closely related to glomerular filtration rate (eGFR) and proteinuria. Because diabetic patients are often accompanied by varying degrees of renal function damage, if the study does not fully correct the renal function factors, the decrease of Klotho level may reflect the independent effect of renal function decline rather than the disease itself, thus overestimating or underestimating its true association with diabetes and complications. Therefore, future studies should simultaneously assess renal function, proteinuria and other influencing factors to more accurately interpret the relationship between Klotho and disease risk and progression. Third, the dose-response relationship may exhibit nonlinear or threshold effects. Some epidemiological and animal studies suggest that the relationship between Klotho and disease risk is not a simple linear, but may be a U-shaped or L-shaped curve. For example, low levels of Klotho significantly increase the risk of kidney or cardiovascular damage, while whether high levels bring potential side effects is still unclear, which adds complexity to the determination of clinical intervention doses and safety ranges. In addition, different subtypes of Klotho (α-Klotho, β-Klotho) may have differences in tissue distribution and physiological functions, and their combined effects, tissue-specific effects and long-term exposure effects still need to be further studied. Fourth, clinical trial evidence is still limited. So far, most studies have focused on cross-sectional observations or small-scale clinical cohorts, and there is still a lack of large-sample, prospective, randomized controlled intervention trials to directly verify whether increasing Klotho levels can improve the clinical outcomes of diabetic patients. The efficacy and safety of Klotho intervention are not yet clear, especially in different stages of disease (early diabetes, microvascular complications, or advanced kidney disease/heart failure) and different populations (age, gender, genetic background). Future research should combine strategies such as gene therapy, recombinant protein or Klotho-derived peptide to evaluate dose-response relationship, efficacy persistence and potential side effects, so as to promote its transformation to clinical application. In addition, multi-factor joint analysis remains to be carried out. Diabetes and its complications are complex diseases with multi-mechanism and multi-factor interactions. The combined detection of Klotho and other biomarkers (such as FGF23, NT-proBNP, VEGF, inflammatory factors, etc.) may provide more accurate risk stratification and disease progression monitoring. By integrating serum/urine levels, genetic polymorphisms, imaging indicators and clinical parameters, a comprehensive risk prediction model can be established to provide a more reliable basis for precise intervention. On the whole, due to the limitations of current methodology and clinical transformation, key obstacles still need to be overcome to make Klotho move from laboratory research to clinical application, including detection standardization, confounding factor correction, dose effect clarification and high-quality clinical trial verification With the continuous optimization of detection techniques and intervention strategies, Klotho is expected to develop from a ‘ potential molecular factor ‘ to an operable clinical tool for early warning of diabetes, prevention and control of complications, and individualized intervention.

## 8 Conclusion and prospect

On the whole, the research of Klotho in diabetes and its complications is showing a trend of gradual transformation from basic mechanism to clinical application, but there are still multiple challenges in this process. Laboratory studies have clearly shown that Klotho plays a protective role in kidney, cardiovascular and retina through anti-oxidation, anti-inflammation, anti-fibrosis and regulation of metabolic signaling pathways (such as Nrf2, Wnt/β-catenin, PI3K/Akt, TGF-β/Smad, etc.), providing a theoretical basis for the intervention of diabetes-related organ damage. Animal experiments have further verified the protective effect of Klotho. For example, in diabetic mouse models such as Akita and db/db, exogenous Klotho or Klotho-derived peptides can reduce glomerular hypertrophy, fibrosis and oxidative stress, while improving myocardial hypertrophy and retinal ganglion cell damage. Nevertheless, the implementation of clinical applications still faces several key bottlenecks. The first is the problem of detection and standardization. In the existing clinical studies, the determination methods of sKlotho are not uniform, including the differences in sensitivity and specificity of different ELISA kits, which makes it difficult to directly compare the cross-study results. At the same time, the normal reference range and disease threshold of serum or urine sKlotho are not yet clear, which limits its reliability as a diagnostic or risk prediction tool. Furthermore, considerable variability exists across different study populations. Circulating sKlotho levels are influenced by age, sex, ethnicity, genetic background, renal function, dietary patterns, and inflammatory status, leading to substantial heterogeneity in reported findings. These demographic and biological differences may partly explain why population-based cohorts, hospitalized patients, and region-specific groups often yield inconsistent results. Therefore, future clinical applications of Klotho will require population-specific reference ranges, stratified analyses, and multicenter validation to ensure the robustness and generalizability of conclusions. The second is the lack of causal verification. Although animal models and *in vitro* experiments have shown that exogenous Klotho can improve diabetes-related organ damage, clinical evidence is still lacking, especially the improvement of long-term outcomes (such as deterioration of renal function, incidence of heart failure, and decreased vision) has not been systematically verified. Dose, route of administration, timing of intervention, and differences in response among different patient groups still need to be further clarified. Thirdly, the potential of multi-factor integration has not been fully explored. Diabetes and its complications are complex diseases with multiple mechanisms and multiple factors. The combined detection of Klotho and other biomarkers (such as FGF23, NT-proBNP, VEGF, inflammatory factors, etc.) may better reflect disease progression and risk stratification. The construction of a multi-index risk model containing Klotho is expected to provide a more reliable tool for precise intervention and individualized management. In addition, Klotho gene polymorphisms (such as G-395A, C1818T, KL-VS) are potentially associated with the susceptibility to diabetes and its complications, suggesting that individualized risk assessment and intervention can be carried out in the future in combination with genetic background. With the standardization of detection methods and the optimization of intervention strategies (such as gene therapy, recombinant proteins or derived peptides), Klotho is expected to develop from a ‘ theoretical potential factor ‘ to an operable clinical tool and play a central role in early warning, prevention and control of complications and individualized intervention of diabetes. Future research should integrate basic mechanisms, animal experiments and clinical verification, and focus on different disease stages, different beneficiary populations and long-term efficacy, so as to accelerate the process of Klotho to clinical transformation.
